# O3 to O1 Phase Transitions in Highly Delithiated NMC811 at Elevated Temperatures

**DOI:** 10.1021/acs.chemmater.3c00307

**Published:** 2023-06-20

**Authors:** Zachary Ruff, Chloe S. Coates, Katharina Märker, Amoghavarsha Mahadevegowda, Chao Xu, Megan E. Penrod, Caterina Ducati, Clare P. Grey

**Affiliations:** † Yusuf Hamied Department of Chemistry, University of Cambridge, Cambridge CB2 1EW, U.K.; ‡ The Faraday Institution, Quad One, Harwell Science and Innovation Campus, Didcot OX11 0RA, U.K.; § Department of Materials Science and Metallurgy, University of Cambridge, 27 Charles Babbage Road, Cambridge CB3 0FS, U.K.

## Abstract

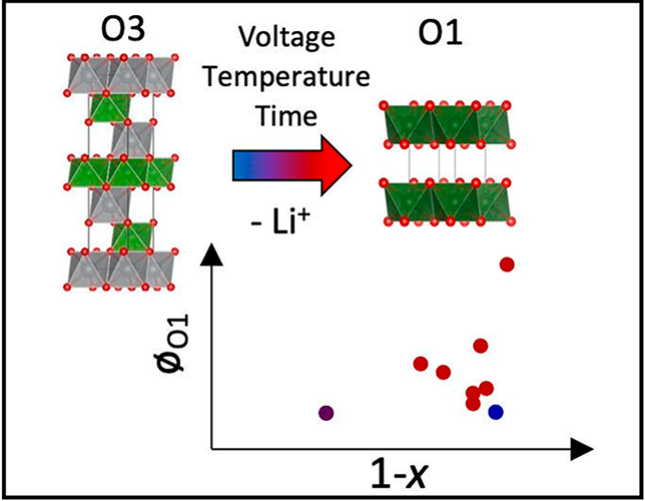

Nickel-rich layered oxide cathodes such as NMC811 (Li_*x*_Ni_0.8_Mn_0.1_Co_0.1_O_2_) currently have the highest practical capacities of cathodes used commercially, approaching 200 mAh/g. Lithium is removed from NMC811 via a solid-solution behavior when delithiated to *x*_Li_ > 0.10, maintaining the same layered (O3 structure) throughout as observed via operando diffraction measurements. Although it is possible to further delithiate NMC811, it is kinetically challenging, and there are significant side reactions between the electrolyte and cathode surface. Here, small format, NMC811-graphite pouch cells were charged to high voltages at elevated temperatures and held for days to access high states of delithiation. Rietveld refinements on high-resolution diffraction data and indexing of selected area electron diffraction patterns, both acquired *ex situ*, show that NMC811 undergoes a partial and reversible transition from the O3 to the O1 phase under these conditions. The O1 phase fraction depends not only on the concentration of intercalated lithium but also on the hold temperature and hold time, indicating that the phase transition is kinetically controlled. ^1^H NMR spectroscopy shows that the proton concentration decreases with O1 phase fraction and is not, therefore, likely to be driving the O3–O1 phase transition.

## Introduction

Nickel-rich layered oxide cathodes, such as lithium nickel manganese cobalt oxide (NMC) and lithium nickel cobalt aluminum oxide (NCA) cathodes, currently have some of the highest practical capacities used commercially, approaching 200 mAh/g.^1^ However, the theoretical capacities of these materials can exceed 270 mAh/g if one lithium ion per formula unit is removed. Although it is possible to extract some of this additional capacity, further delithiation is kinetically challenging and leads to undesirable side reactions such as oxygen release,^2^ electrolyte oxidation,^3^ transition metal (TM) dissolution,^4^ and reduced surface phases.^5^ Both the reduced surface phases and the poor bulk lithium diffusivity in the cathode particle^6^ make further delithiation even more difficult. Nonetheless, the structural stability of highly delithiated nickel-rich layered oxides is of interest both from a fundamental perspective and for the practical understanding of why further capacity cannot be extracted.

**Figure 1:**
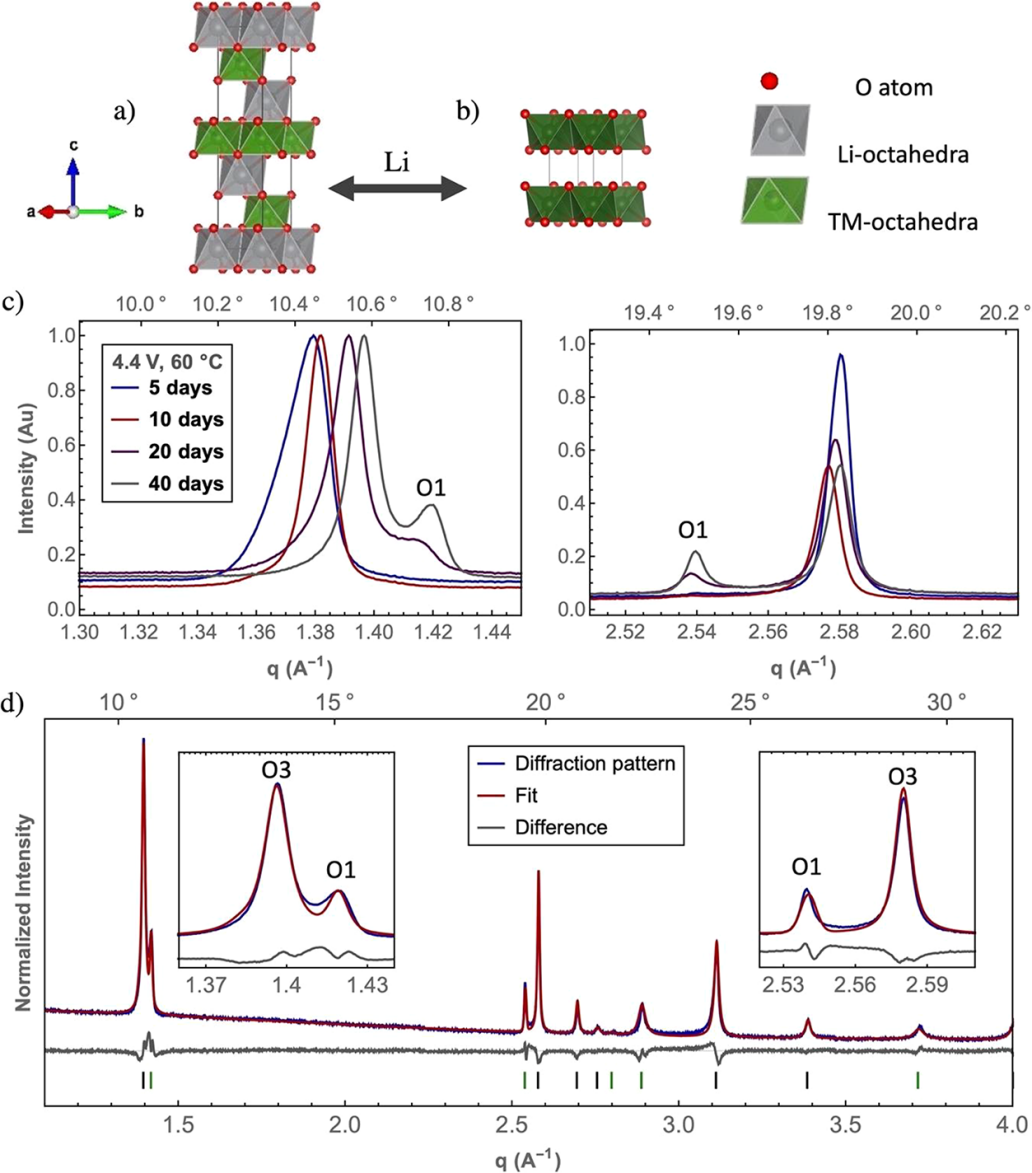
Structural model of O3 (a) and O1 (b) LiTMO_2_ phases (TM = Co, Ni, etc.). The Li and TM octahedra are shown in green and gray, respectively, with oxygen atoms represented by the red spheres. Diffraction patterns of NMC811 cathodes extracted from NMC811/graphite cells held at 4.4 V at 60 °C for 5, 10, 20, and 40 days (c). The new reflections at approximately 1.42 and 2.54 Å^–1^ are indexed to the O1 phase. Refinement of the cathode electrode (held at 4.4 V and 60 °C for 20 days) with both the O3 (black ticks) and O1 (green ticks) phases present (d). Insets in (d) show magnified portions of the patterns of particular interest.

Due to kinetic and thermodynamic limitations, NMC811 (LiNi_0.8_Mn_0.1_Co_0.1_O_2_) is typically cycled reversibly between a lithium concentration *x* (in Li_*x*_Ni_0.8_Mn_0.1_Co_0.1_O_2_) of ∼0.95 and 0.25, giving a practical capacity of ∼190 mAh/g when paired with a graphite anode. The upper limit for *x* is set by two phenomena: (i) approximately 5% of the lithium is typically consumed at the anode to form the solid electrolyte interphase (SEI) on graphite, and (ii) it is difficult to reinsert all the lithium due to the poor lithium mobility as *x* approaches 1.0.^7^ The capacity limitations are not due to any structural phase transitions, as NMC811 maintains the O3 layered structure (*R*3*m* space group, α-NaFeO_2_ structure, Figure 1a) between 1.0 > *x* > 0.10^6,8^ with the layer spacing (and *c*-lattice parameter) first increasing and then decreasing. This solid–solution behavior is in contrast to both lithium nickel oxide (LNO)^9^ and lithium cobalt oxide (LCO),^10,11^ which undergo a number of hexagonal and monoclinic phase transitions from the starting O3 phase upon delithiation. Note that the O3 (Delmas) nomenclature indicates that the lithium ions are octahedrally coordinated (O) and that three layers of edge-sharing TM-O_6_ octahedra sheets, alternating with layers of lithium ions, are needed to define a unit cell.^12^

Below approximately *x* = 0.15, Amatucci et al.^11^ showed that LCO undergoes a two-phase reaction from a monoclinic O3 to the O1 phase, which is accompanied by a sharp decrease in the *c*-lattice parameter. This transition is associated with a shearing of the lattice such that the TM octahedra go from edge-sharing with the Li octahedra to face-sharing, and the number of repeating TM-O_6_ layers needed to define the unit cell is reduced to a single layer (Figure 1b). They concluded that the O1 phase is formed when all the lithium is removed from the lattice (to form CoO_2_); the stability of this phase was later supported by first-principles calculations of Van der Ven et al.,^13,14^ who calculated an energy gap of 40 meV per formula unit favoring the O1 phase over the fully delithiated O3 phase. Croguennec et al. used operando diffraction to show that LNO also forms the O1 phase at high states of delithiation, but the transformation is not complete, with residual O3 phase remaining even after holding at elevated voltages (4.45 V vs Li/Li^+^) for 815 h (20.4 days, at room temperature).^9,15,16^ This kinetically slow and incomplete transition is likely due to the combination of the smaller energy gap for the transition (calculated to be only 7 meV per formula unit^17^) and the presence of antisite defects, where Ni^2+^ substitutes for Li^+^ in the lithium layers, which prevents layer gliding.^9^

In both NMC^18^ and NCA,^19^ the O1 phase has been observed locally at the electrolyte/cathode interface: thin surface phases were identified using selective area diffraction measurements (SAED) in high-resolution transmission electron microscopy (TEM) on cathodes that had been pushed to high potentials at elevated temperatures. However, to our knowledge the O1 phase has not yet been observed in nickel-rich layered oxide cathodes such as NMC811 in sufficient phase fractions to be identified using bulk sensitive techniques such as X-ray diffraction (XRD).

In this work, we show that NMC811 does undergo a partial bulk transition from the O3 to the O1 phase when held at elevated potentials and temperatures for days. The O1 phase is identified by performing Rietveld refinements utilizing XRD data and indexing of SAED patterns from cathodes extracted from small format NMC811-graphite pouch cells. We explore the kinetics of the transition by delithiating the cathodes under a range of conditions and show that the transition is reversible upon discharge. ^1^H and ^7^Li nuclear magnetic resonance (NMR) spectroscopy was also performed and is coupled with results from the Rietveld refinements and composition analysis for different experimental conditions to gain insights into the mechanism of the phase transitions.

## Experimental Methods

### Cell Materials

Multilayer pouch cells with a nominal capacity of 200 mAh were purchased dry and sealed from LiFun. The NMC811 powder was purchased from Targray and provided to LiFun, while the synthetic graphite powder was Kaijin AML-400. Both the cathode and anode of the pouch cells are double-side-coated, and the single-side loadings of the active materials are 18.0 mg/cm^2^ (96.4% active material) for cathode and 13.0 mg/cm^2^ (94.8% active material), giving a negative-to-positive capacity ratio (N/P) of approximately 1.3. Single-crystalline pouch cells consisting of a LiNi_0.83_Mn_0.1_Co_0.07_O_2_ cathode and the same graphite anode were also tested with a loading of 16.7 mg/cm^2^ (95.5% active material).

The pouch cells were opened and dried further at 70 °C overnight in a vacuum oven and then filled with 0.9 mL of LP57 electrolyte (1 M lithium hexafluorophosphate in a 3:7, ethylene carbonate to ethyl methyl carbonate) and vacuum-sealed in a dry room before electrochemical testing.

### Electrochemical Cycling and Sample Preparation

After filling and sealing, the pouch cells were placed in climatic chambers and charged to 1.5 V and held for 24 h. The cells then underwent two formation cycles cycling performed between 2.5 and an upper-cut voltage (UCV) of 4.2 V at a rate of C/20, where 1 C is the amount of current required to fully charge the cells to 4.2 V in 1 h. The cells subsequently were charged to the UCV at a rate of C/10 and then held at the UCV for a given period of time (days). After the voltage hold, the cells were disassembled in the charged state in an argon glovebox to recover the conditioned cathodes. The cathodes were rinsed with dimethyl carbonate and dried under vacuum before the cathode powders were scraped off the aluminum current collectors for further characterization. Voltage and current profiles from the cell cycling can be seen in Figure S1.

### Compositional Analysis

The cathode particles were digested overnight by using aqua regia prepared from trace element grade nitric and hydrochloric acids (Fisher Scientific). After letting the conductive carbon sediment, the supernatant consisting of the digested cathode powder was diluted with 2% nitric acid for the inductively coupled plasma optical emission spectroscopy (ICP-OEMS, Thermoscientific) measurement. The concentration of a given element in the solution was determined by comparing the emission of the sample solutions to a calibration line generated from a concentration series made from a multielement standard (VWR, Aristar) at each wavelength of interest. The emission wavelengths were selected such that there was no interference from other elements in the sample, elements in the standard, or the matrix solution (2% nitric acid). The lithium concentration (*x*) in the cathode particles was calculated based on the assumption of 1 mol of transition metal (Li_*x*_Ni_0.8_Mn_0.1_Co_0.1_O_2_) per formula unit. Where it is assumed that there is no lithium in the O1 phase, *x* in the O3 phase was calculated by
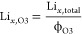
1where ϕ_O3_ is the phase fraction of the O3 phase (see the Supporting Information).

### Diffraction Measurements and Refinements

High-resolution X-ray diffraction data were obtained at the I11 beamline at the Diamond Light Source, UK. Cathode powders were ground and packed into 0.5 mm external diameter borosilicate capillaries (Capillary Tube Supplies Ltd.), which were sealed with epoxy in an argon glovebox. XRD patterns were measured using a position-sensitive detector (PSD). The wavelength and peak shape parameters were refined against a Si standard and are indicated for each fit. The beam energy was approximately 15 keV (∼0.827 Å). Rietveld refinements were performed using TOPAS Academic (ver. 6.0).

Significant asymmetry in the peak shapes of the O3 phase (00*l* reflections in particular) was observed for different samples. In order to obtain the best estimate of the average *a* and *c* lattice parameters, we employed a multiphase fit to account for the distribution of unit cell parameters, similar to that used by Orr et al.,^20^ to describe core–shell gradients. Ten O3 phases were used, and the structure of each was identical, except for the lattice parameters. The lattice parameters of each phase were linked by the parameters σ_*a*_ and σ_*c*_ as follows:
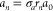
2
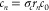
3*a*_1_...*a*_10_ are linked linearly; *r*_1_ = 0.1 and *r*_*n*_ = *r*_*n*__–1_ + 0.1. Each phase has a freely refinable scale factor. The scale factors and resulting lattice parameters were used to determine a weighted average lattice parameter and a weighted standard deviation for each lattice parameter. All other parameters were refined for the phases collectively. The lithium occupancy was fixed to be that measured by ICP. Other refined parameters included the oxygen *z* position, anisotropic displacement parameters for the transition metal site, and particle size broadening parameters (∼200 nm diffracting domain size, which corresponds approximately to the size of the primary particles of NMC811).

### Electron Microscopy and Diffraction Measurements

A focused ion beam scanning electron microscope, FEI Helios NanoLab FIB/SEM, was used to prepare and extract the TEM lamella with a final cross section thickness of approximately 100–150 nm. A transmission electron microscope Thermo Scientific (FEI) Talos F200X G2 was operated at 200 kV to acquire bright-field transmission electron microscopy (BF-TEM) images and SAED patterns from this lamella. CrystalMaker and SingleCrystal software were used to analyze and simulate the single crystal kinematic electron diffraction patterns.

### Solid-State NMR Measurements

Solid-state NMR measurements were performed on cathode powders packed into 1.3 mm NMR rotors (sample masses between 5.6 and 6.1 mg). The NMR spectra were acquired on a 7.05 T (300 MHz ^1^H Larmor frequency) Bruker Avance NMR spectrometer, using a 1.3 mm double-resonance magic-angle spinning (MAS) NMR probe (Bruker). The samples were spun at 55 kHz MAS frequency. ^1^H and ^7^Li NMR spectra were acquired using a Hahn echo pulse sequence with a total echo delay of two rotor periods (36.4 μs). For the ^1^H NMR spectra, radio-frequency (RF) pulses were applied at an RF field strength of 188 kHz, and 40480 transients were acquired with a recycle delay of 5 ms. A spectrum with identical settings was recorded with an empty probe and subtracted from the ^1^H spectra of the samples to remove ^1^H signals coming from the probe. For ^7^Li spectra, RF pulses were applied at an RF field strength of 167 kHz, and 40480 transients were acquired with a recycle delay of 30 ms.

## Results

### XRD Measurements and Rietveld Refinements

To explore the structural stability of NMC811, small multilayer pouch cells with graphite anodes underwent two formation cycles and were then charged and held at elevated voltages (4.4 V vs graphite and above) and temperatures (25, 40, or 60 °C) for different periods of time (days) to achieve states of delithiation that are often kinetically inaccessible. The cells were then disassembled in the charged state, and synchrotron XRD patterns were taken of the cathodes. The lithium concentration in the cathode was measured using ICP and the current passed during the voltage holds was also recorded (Table S1). After holding the pouch cells at 4.4 V for 20 days at 60 °C, new reflections in the XRD patterns can be observed at 1.42 and 2.54 Å^–1^ that were not present in the cells held for 5 and 10 days (Figure 1c). The intensity of the new reflections was greatest for the cell held for 40 days. The positions of the new reflections agree well with experimental results for the O1 phase in LCO^10,11^ and LNO^9,15^ and not with reflections from, for example, spinel or rock-salt surface phases. We note here that all the voltages quoted in this article are for NMC811/graphite cells, where the graphite is lithiated to stage 1 (70 mV vs Li/Li^+^, verified by XRD, Figure S2). Therefore, 70 mV should be added to these voltages to compare to works where the cathode potentials are reported vs Li/Li^+^ (i.e., a voltage of 4.40 V in our NMC/graphite cells corresponds to 4.47 V vs Li/Li^+^).

To verify that the new reflections can indeed be assigned to the O1 phase, Rietveld refinements were performed using the diffraction data (Figure 1). Significant asymmetry in the peak shapes of the O3 phase (00*l* reflections in particular) was observed for different samples. To obtain the best estimate of the average *a-* and *c-*lattice parameters, we employed a multiphase fit to account for the distribution of unit cell parameters, similar to that used by Orr et al.^20^ for describing core–shell gradients. In this way a more accurate (weighted) average lattice parameter was obtained to describe the O3 phase. The O1 phase was modeled using a single phase with symmetry *P*3*m.* The refinements assumed that the O1 phase was completely devoid of lithium because the O1 *c-*lattice parameter did not change significantly with the overall (average) lithium content of the cathode as determined from ICP (Figure 3). The average lithium content of the cathode material therefore was used to constrain the lithium content in the O3 phases. A representative refinement for a sample held at 4.4 V at 60 °C for 20 days can be seen in Figure 1d. Our multiphase refinement can capture the asymmetry of the O3 peak and results in a fit with *R*_wp_ = 5.65%. We note here that there is residual intensity between the O3 003 reflection and the O1 001 reflection (see the inset at *q* ∼ 1.41 Å^–1^) that is not captured by our model, which could arise from stacking faults.^16^ Full stacking fault analysis is an avenue for future work.

### TEM

**Figure 2:**
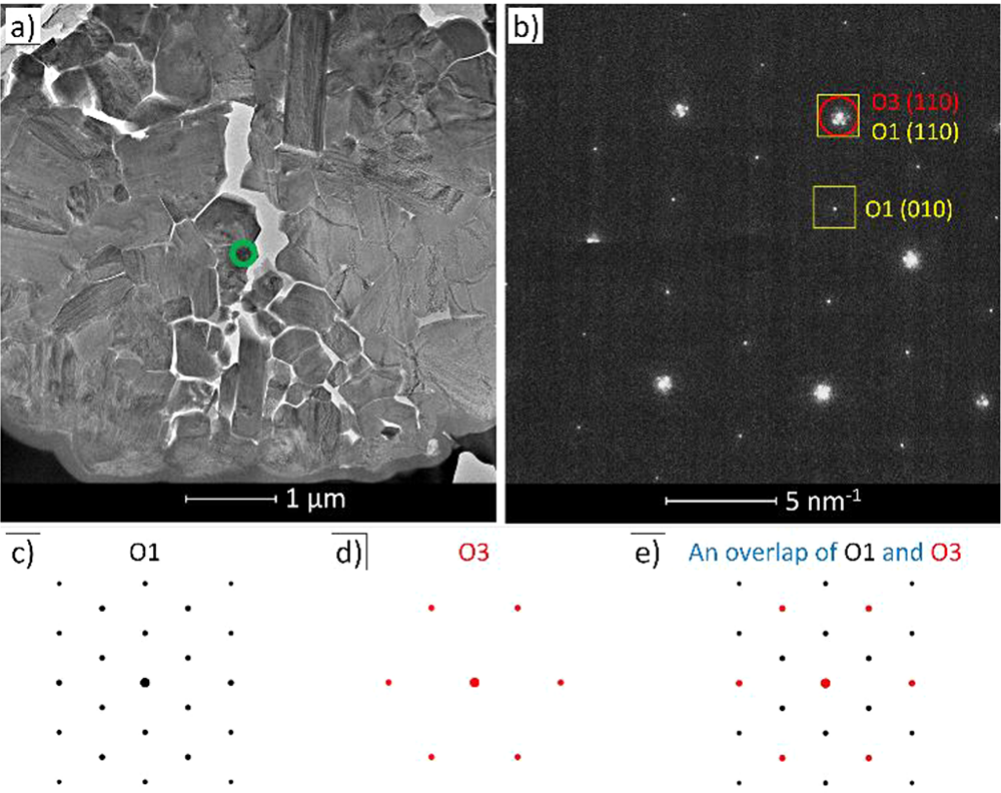
BF-TEM cross-sectional image of secondary particle of NMC811 from a cell held at 4.4 V and 60 °C for 40 days (*x* = 0.05, ϕ_O1_ = 24%) (a). SAED pattern (b) from the region marked by a green circle in (a), which is indexed as an [001] projection of the NMC811 lattice. The pattern shows additional reflections assigned to the O1 phase (b). O3 and O1 reflections in (b) are highlighted by a red circle and a yellow square, respectively. Simulated electron diffraction patterns of O1 (c) and O3 (d) and an overlap of these two patterns (e) along the [001] orientation. The simulated patterns (c–e) are rotated by 90° counterclockwise about the out-of-plane axis to aid the eye in comparing them with the experimental pattern (b).

The presence of the O1 phase also was verified by TEM. Bright-field (BF) TEM was used to image the cross section of a secondary particle of NMC811 prepared by focused ion-beam milling and extracted from the cell held at 4.4 V and 60 °C for 40 days (Figure 2a).^21^ SAED was performed on an area of approximately 200 nm in diameter (green circle in Figure 2a), including the specific example shown in Figure 2b, which corresponds to a [001] projection of the NMC811 lattice. This orientation was chosen to simplify the identification of the O1 phase based on its (*hkl*) reflections, as shown in the simulated electron diffraction patterns (Figure 2c,d). An overlap of the simulated patterns of the two phases is shown in Figure 2e, which is a close match to the experimentally obtained electron diffraction pattern. The indexed reflection in Figure 2b (110) contains contributions from both the O3 (red circle) and O1 (yellow squares) phases. However, the other reflection highlighted in Figure 2b can only be assigned to the O1 phase ((010) reflection). Additional indexed simulated electron diffraction patterns for the two phases are shown in Figure S8.

The primary (110) reflection has additional adjacent secondary reflections, suggesting that the electrons underwent double diffraction^22,23^ as they traveled through the 100–150 nm thick cross section, consistent with the presence of two overlapping crystalline domains with different *d*-spacings in the area selected (here O1 and O3). A qualitative comparison of the intensities would suggest that the O1 phase is less dominant in this region. We note that the simulated single crystal patterns do not account for these additional secondary reflections.

### Kinetics, Reversibility, and Self-Discharge

**Figure 3:**
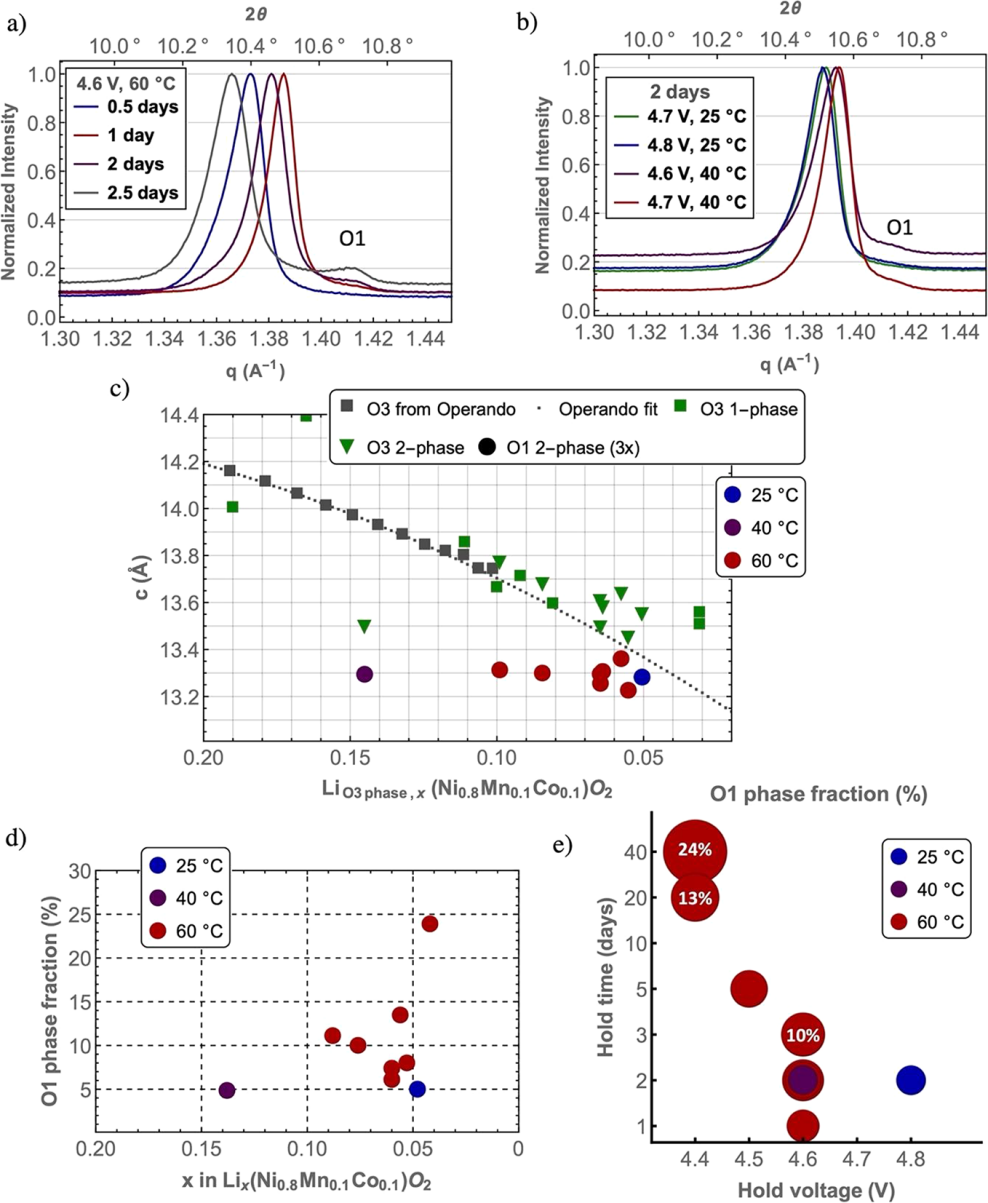
XRD (λ= 0.827 Å) patterns for cells held at 4.6 V at 60 °C (a). XRD patterns for cells held at 4.7 and 4.8 V at 25 and 40 °C for 2 days (b). Comparision of the *c*-lattice parameters of the O1 and O3 phases obtained from Rietveld refinements for all the sample studied in this work . These are plotted on the same chart as the *c*-parameters obtained from refinements using operando diffraction data. An extrapolation of the operando-derived cell parameters using a spline fit against the lithium content in the O3 phase is shown (gray dashed line) (c). The lithium content for the two-phase samples is calculated from the ICP data assuming there is no lithium in the O1 phase, i.e., lithium is only present in the O3 phase. The O1 phase fraction from the Rietveld refinements is plotted against the lithium content in the NMC811 material for all the samples studied in this work prepared at different temperatures (and voltages) in (d). The same data are plotted in (e) as a bubble chart showing the O1 phase fraction dependence on the hold voltage, temperature, and time, where the area of the bubble is proportional to the phase fraction; the phase fraction (%) is marked on three bubbles. The same temperature color scheme applies to (c–e).

The O1 phase also was observed for cells tested in other conditions. For example, for cells held at 4.6 V at 60 °C, the O1 phase was observed after holding for only 1 day with the peak intensities increasing further after holding for 2.5 days (Figure 3a). Holding for longer than 2.5 days at 4.6 V and 60 °C caused the pouch cells to burst due to the extreme gassing at these stressed conditions. To allow comparison between different samples, the refined weighted average of the *c*-lattice parameter of the O3 phase and the *c* parameter for the O1 phase (multiplied by 3 to account for the single vs three layers of the O1 vs O3 phase per unit cell) vs the lithium concentration (*x*) in the O3 phase are plotted in Figure 3c, for cells held at all conditions (voltage, time, and temperature) explored in this work (Table S1 for tabulated values). For reference, lattice parameters extracted from refinements of an operando measurement on the same cathode powder^6^ are plotted along with an extrapolated spline fit of the operando data. The *c*-lattice parameters of the O3 phases both in electrodes where there is only a single phase (i.e., in cases where the O3 structural model provided a good fit to the data and the O1 phase fraction, ϕ_O1_, was less than 5%) and in electrodes containing two phases (both the O3 and O1 structural models required, ϕ_O1_ ≥ 5%) follow the operando data, a decrease in the *c*-lattice parameter being seen with decreasing lithium content (at these high states of charge). For the two phase samples, it was assumed in the data points shown in this plot that all of the lithium is present in the O3 phase (no lithium is in the O1 phase). The *c*-lattice parameter vs *x* is in worse agreement with the operando measurement when both the O3 and O1 phases are assumed to have the same Li content (for comparison see Figure S4). The *c*-lattice parameter of the O1 phase is invariant with the average lithium content of the cathode, supporting the assumption that the O1 phase has a fixed lithium concentration (*x*) approaching zero.

To explore the kinetics of the O3 to O1 phase transition, we consider the cells that were held at even higher voltages, 4.7 and 4.8 V, but at lower temperatures, 25 and 40 °C (Figures 3b and S3). At these conditions, the average lithium content in the cathodes was below *x* < 0.05, but the strong 001 and 100 reflections f from the O1 phase were not present. In fact, no significant O1 phase (ϕ_O1_ < 5%) was found at the 25 °C hold temperature until the voltage was increased to 4.8 V. At 40 °C, ϕ_O1_ was somewhat higher, but still significantly less than at 60 °C. The O1 phase fraction is plotted for all the samples prepared in this work using two different graphical representations (Figure 3d,e) highlighting the ϕ_O1_ dependence on hold voltage, temperature, and time. These experiments show that a high average delithiation of the NMC particles alone is not sufficient for driving the phase transition, suggesting a kinetic barrier to nucleating and/or growing the new phase.

**Figure 4:**
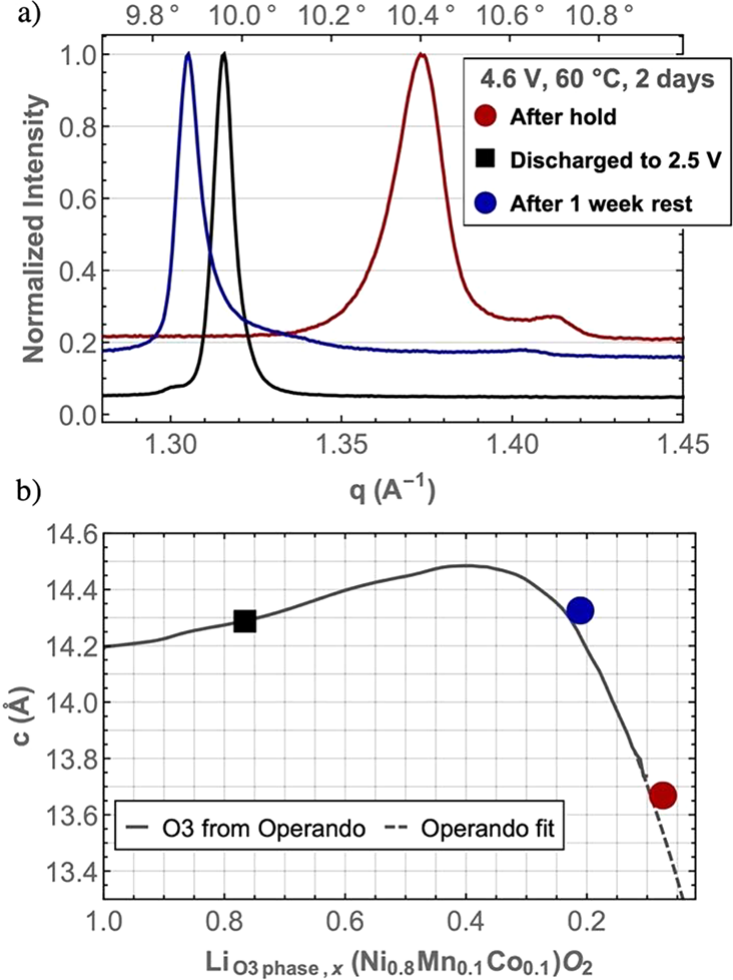
Diffraction patterns (λ= 0.827 Å) of cathodes extracted from cells held at 4.6 V and 60 °C for 2 days right after the voltage hold, after discharging to 2.5 V and after resting the cell at open circuit voltage for 1 week at 60 °C (a). *c*-lattice parameters of the O3 phase from the same cathodes plotted against the operando data vs their lithium content determined from ICP assuming there is no lithium in the O1 phase (b).

To determine if the O3 to O1 phase transition is reversible, three cells were held at conditions where it was previously demonstrated that a significant fraction of the O1 phase is formed (4.6 V and 60 °C for 2 days). Afterward, one cell was disassembled immediately, one cell was discharged to 2.5 V, and one cell was left to rest at open circuit voltage (OCV) for a week at 60 °C before disassembly; ex-situ analysis was then performed (Figure 4a). For the cell disassembled after discharging, no reflections from the O1 phase were observable, and the refined *c*-lattice parameter of the O3 phase returned to values consistent with the operando measurements (Figure 4b). The voltage of the cell charged and then left at OCV dropped slowly to 4.06 V (Figure S5) due to self-discharge, the (average value of the) *c*-lattice parameter of the O3 phase following the operando curve (vs *x*, Figure 4b), indicating that self-discharge leads to a relithiation of the O3 phase. The 003 reflection from the O3 phase of this sample also became highly asymmetric with a broad tail tending to high *q* values, indicating the compositional heterogeneity (in *x*) of the O3 phase. The intensities of the O1 reflections were somewhat smaller (ϕ_O1_ = 10% to ϕ_O1_ = 8%). However, the difference in the ϕ_O1_ maybe due to experimental variability in ϕ_O1_ before self-discharge because refinements on the two different samples held at 4.6 V and 60 °C for 2 days led to somewhat different values for ϕ_O1_ (7% vs 10%). Therefore, the O3 to O1 phase transition is reversible upon discharge, but self-discharge did not result in a transition of the sample back to the O3 phase.

We also have delithiated “single crystal” Li_*x*_Ni_0.83_Mn_0.1_Co_0.07_O_2_ (i.e., a sample composed of largely isolated micrometer sized NMC particles) at similar conditions used here and identified the O1 phase (Figure S7) at similar phase fractions. However, quantifying phase fractions in the single crystal samples was more challenging due to the large heterogeneity of the *c*-lattice parameter and, therefore, lithium content within the O3 phase across the sample.

### ^1^H and ^7^Li Solid-State NMR Spectroscopy

**Figure 5:**
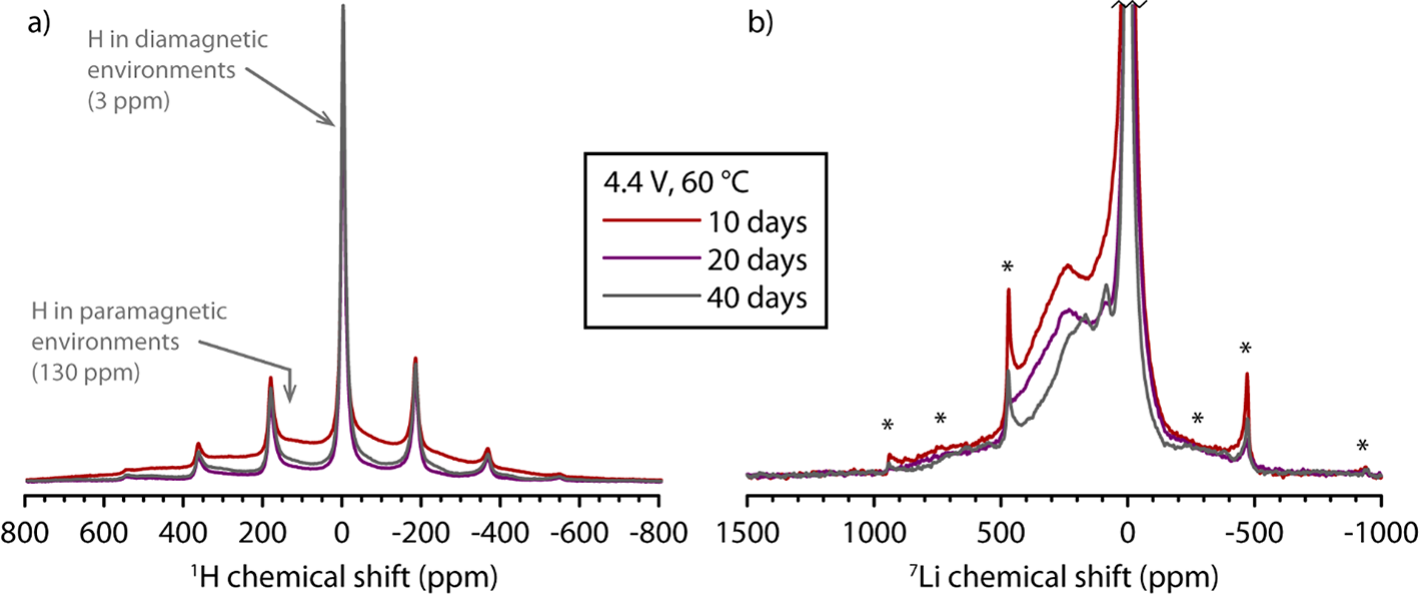
Solid-state NMR spectra of cathodes extracted from cells held at 4.4 V and 60 °C for 10 days (*x* = 0.08, ϕ_O1_ = 0%), 20 days (*x* = 0.06, ϕ_O1_ = 13%) and 40 days (*x* = 0.05, ϕ_O1_ = 24%). The cathode materials for the NMR measurements were extracted from the same cells as in Figure 1c. (a) ^1^H Hahn-echo NMR spectra. The isotropic signals from protons in diamagnetic and paramagnetic environments are indicated by arrows; all other resonances are spinning sidebands (see Figure S5a for a projection-MATPASS spectrum). (b) ^7^Li Hahn echo NMR spectra, with spinning sidebands are indicated by asterisks (∗). All spectra were recorded at 7.05 T magnetic field strength and 55 kHz magic-angle spinning frequency. The spectra are normalized by sample weight and number of scans.

In studies where NMC has been chemically delithiated, it has been suggested that protons may facilitate phase transformations from the O3 to the P3 phase, a phase that is stabilized by hydrogen bonding between the TM-O_6_ layers.^24^ To determine the impact of the protic species generated during the voltage hold, e.g., via electrolyte degradation,^3^^1^H MAS NMR was performed on cathodes from the same cells as evaluated in Figure 1c, specifically cells held at 4.4 V at 60 °C for 10, 20, and 40 days (Figure 5a). The ^1^H NMR spectra show an intense and relatively sharp signal centered at ∼3 ppm, originating from residual electrolyte, other diamagnetic species on the cathode surface, and the binder. In addition, a much broader signal is observed at ∼130 ppm (see also Figure S6a). The very broad line width of this resonance, its shift being far outside the conventional ^1^H chemical shift range (0–20 ppm), as well as its extremely short longitudinal, spin–lattice (*T*_1_) relaxation time (≤1 ms) strongly suggests that these protons are close to paramagnetic species (Ni^2/3+^ and Mn^4+^)^25^ and therefore could be located in the lithium layers as well as in defects or edge sites. Similarly large, positive (^2^H) hyperfine shifts have been seen for NiOOD^26^ and acid-leached Li_2_MnO_3_.^27^ However, the proton concentration is highest in the sample held for 10 days and considerably lower in the other two samples. Given that the O1 phase is only observed in the 20 and 40 day samples, it is unlikely that proton intercalation is driving the transition to the O1 phase, unless there is a two-step process where proton insertion nucleates or triggers layer shearing and then the protons subsequently are removed or remain at defect sites. Therefore, although there are high proton concentrations in these samples, which we attribute to the products of electrolyte oxidation, the protons are not believed to be associated with the O3 to O1 transformation. No clear signature of a P3 phase was seen by XRD, a phase that was seen during chemical delithiation, albeit for lower Ni-content NMCs, and under conditions that allow for rapid delithiation (namely low antisite mixing and high surface areas).^24^ We note that the kinetics of the electrochemical delithiation process used here differ significantly from those of a chemical delithiation, the latter being closer to delithiation at a fixed high-voltage hold or short circuit, which may, in part, account for differences in phase fractions seen between the two approaches.^24^

^7^Li MAS NMR spectra were acquired on the same samples to further investigate the structure of highly delithiated NMC811 (Figure 5b, full discussion in the Supporting Information). The ^7^Li signal intensity decreases with increasing hold time, in agreement with the lithium concentration obtained from the ICP measurements. The dominant signal is observed at ∼0 ppm (see Figure S6b for full spectra) and can be attributed to Li^+^ ions in diamagnetic environments. These occur both in surface species and in octahedral sites in the lithium layers of NMC that are surrounded only by diamagnetic TM ions. The latter can be expected to be the dominant environment for Li^+^ ions in NMC at these states of delithiation, as diamagnetic TM ions (Ni^4+^ and Co^3+^) account for at least 80% of the TM ions. Under the assumption that nickel oxidation to Ni^4+^ is complete and that cobalt oxidation to Co^4+^ does not cause an observable shift of the ^7^Li resonance,^28^ the ^7^Li signals at positive ppm values can be explained by the presence of paramagnetic Mn^4+^ around the Li^+^ ions, with one Mn^4+^ neighbor in the first coordination shell of lithium expected to cause a resonance (hyperfine) shift of +250 ppm.^6^ A relatively intense signal is indeed observed at this shift. The additional signal intensity between 250 and 0 ppm may (particularly in the 10 day sample) result from Li^+^ ions which possess a certain degree of mobility between sites with and without one Mn^4+^ neighbor (at 250 and 0 ppm, respectively), leading to an averaged hyperfine shift. However, the collapse of the *c*-parameter and the decreased lithium mobility at this state of charge make this explanation unlikely.^6^ Finally, a comparably sharp signal grows in at ∼88 ppm and becomes more resolved with increasing voltage hold time, which makes it tempting to assign it to a small quantity of Li^+^ ions (<0.5%) in the growing O1 phase. This signal, however, also may come from lithium environments in the O3 phase or in paramagnetic surface species. Potential assignments include Li^+^ ions with either residual Ni^3+^ ions in the second coordination shell or Co^4+^ in the first coordination shell. The presence of Co^4+^ ions in LCO in samples that are approaching, but have not been sufficiently delithiated to undergo, the insulator-to-metal transition,^28^ results in loss of the ^7^Li signal. However, the electronic structure of Co^4+^ surrounded by primarily Ni^4+^ and Mn^4+^ is likely very different. The origin of this peak cannot be confirmed without further experiments and/or calculations.

## Discussion

Having ruled out interactions involving protons as a potential driving force of the O3 to O1 phase transition during electrochemically driven delithiation, we can return to a description where the structural changes are driven by interactions between the TM-O_6_ layers after removal of most or all of the Li^+^. The O3 structure is not energetically favorable on delithiating LCO, but the driving force to form O1 is considerably smaller in LNO, as shown computationally.^17^ While the driving forces favoring O3 and O1 are subtle, the increased covalency of the TM–O bonds will reduce the repulsion between oxide layers.^29^ That any O1 phase is formed in NMC811 indicates that this phase represents a thermodynamic minimum. However, the maximum O1 phase fraction observed here is only 24%. This behavior is similar to LNO, which remains two-phase, even after holding at elevated voltages (unlike delithiated LCO, which can transform fully to the O1 phase). In LNO, Croguennec et al. assigned the incomplete conversion to the 2% concentration of antisite mixing between the nickel and lithium layers:^9,15,30^ even in state-of-the-art LNO samples, approximately 2% occupancy of nickel on the lithium sites is almost always invariably found due to the difficulties associated with preventing antisite mixing and lithium loss.^30^ In support of their hypothesis, the O1 phase transition was completely suppressed in their LNO samples with a 7% concentration of antisite mixing. We have shown elsewhere that the NMC811 powder used here also has approximately a 2% concentration of antisite mixing.^31^ In NMC811, the antisite mixing may also set an upper limit for the conversion to the O1 phase, although we have not neccesarily reached that limit in this work. Furthermore, it is likely that the driving force for the phase transition in NMC811 is small and closer to LNO (calculated to be 7 meV per formula unit)^17^ than LCO (40 meV per formula unit).^13,14^ It also is possible that electrolyte oxidation is constantly reinserting lithium back into the cathode, preventing the cathode from fully delithiating and decreasing the thermodynamic driving force for the phase transformation. Calculations to understand the thermodynamics of the O3 to O1 phase transition in NMC811 and the role of stacking faults, antisite mixing, cation disorder, and the cathode’s transition metal composition would be instructive.

The self-discharge process in the cell left at OCV highlights the reactivity between the electrolyte and cathode surface at these high states of delithiation and elevated temperatures. At these cathode potentials, the electrolyte solvent (ethylene carbonate and ethyl methyl carbonate)^3,32^ is oxidized leading to relithiation of the cathode for charge balance. Moreover, some of the oxidation products also may be acting as redox shuttles^33^ adding further to the leakage current that is always present in these cells. Therefore, cathodes held at these stressed conditions will inevitably—at least without the addition of appropriate additives or surface coatings—undergo self-discharge, during the time between the end of the voltage hold and disassembly in the glovebox, introducing sample variability. For example, it is likely to be the reason why the O3 *c*-lattice parameter is larger for the 2.5 day vs the 2 day samples held at 4.6 V and 60 °C (Figure 3a and Table S1). Operando measurements would be beneficial for eliminating this inherent variability.

Here, we re-emphasize that at the conditions required to delithiate the NMC to form the O1 phase there is significant electrolyte oxidation^34^ and deprotonation of the ethylene carbonate solvent^32,34^ introducing protic species to the cell. In NMC811 cells cycled at similar elevated voltages and/or temperatures in our previous work, numerous degradation products were detected in the ^1^H and ^19^F NMR spectra of the electrolyte,^3^ and large amounts of transition metal deposits were measured at the graphite anode.^4^ The degradation is further evident in the large amount of current passed during the voltage holds (Table S1). For example, during the voltage hold of the cell at 4.4 V and 60 °C for 2 days, 77.9 mAh/g or approximately 41% of the practical cell capacity was passed. The degradation processes of these cells held at stressed conditions will be explored further elsewhere.

The morphology of the NMC811 cathode consists of 10–20 μm secondary particles composed of 100–300 nm primary particles,^8^ so in principle individual primary particles could undergo a phase transition. Further SAED measurements are in progress to map the O1 phase distribution in NMC particles and to study the nucleation and growth of the phase transition. Although the diffraction data does not contain any information about the spatial distribution of the O1 phase, it is likely that it originates at the electrolyte/cathode interface, where the material is expected to be more delithiated.^35^ However, the formation of a thick rock-salt layer on the surface of the particles as the material is cycled or aged at higher voltages may hinder layer shearing. We have previously shown that extended cycling leads to an increase in phase fraction of a component (that we described as a “fatigued phase”) that cannot be fully delithiated: lithium is removed until the *c* parameter, and thus average O–O distances in the *c*-direction are close to that of the rock-salt structure, and no *c*-parameter collapse is seen.^8^ While a rock-salt layer may not necessarily be thick enough to prevent layer collapse of the whole primary particle, it may be sufficient to prevent nucleation of the O1 phase, especially if the driving force for O1 formation is weak. Ikeda et al.^36^ have argued that the phase transition in LNO is suppressed by the reversible Ni migration from octahedral to tetrahedral sites at high states of charge. Given that the migration of Ni from an octahedral site to a tetrahedral site represents the first step in rock-salt formation, this does hint at a possible of role of Ni migration suppressing phase transitions at high states of charge, but evidence, at least in NMC811, suggests that these processes are more commonly found at the surface than in the bulk.

## Conclusion

In summary, we have shown that NMC811 undergoes a bulk O3 to O1 phase transition when held at elevated temperatures and voltages. The phase fraction of the O1 phase is dependent on a combination of the hold voltage, temperature, and time rather than simply the lithium concentration in the material, indicating that this phase transition is kinetically slow. Moreover, the phase transition is incomplete even after holding for 40 days at 60 °C, such that a majority of the sample remains in the O3 phase (ϕ_O1_ = 24%). The transition is fully reversible upon relithiation. High proton concentrations were measured in these samples, consistent with electrolyte degradation; however, the measured increase in proton concentration did not track the increased O1 phase fraction, suggesting that protons do not drive the phase transition. These insights expand the fundamental understanding of the O3 to O1 transition in nickel-rich layered cathodes and may have practical implications as these materials are pushed to high states of delithiation.

## Supporting Information Available

Electrochemistry; table summarizing cell conditions, electrochemical data, ICP measurements and Rietveld refinements; diffraction patterns of graphite anodes; additional high-resolution diffraction data; calculations and plots for adjusting lithium concentrations in O3 phase; voltage and current profiles for cell left at OCV after voltage hold; additional NMR spectra; diffraction measurements on “single crystal” particles; indexed simulated diffraction patterns (PDF)
