# Supplementary material for: O3 to O1 Phase Transitions in Highly Delithiated NMC811 at Elevated Temperatures
Source: Chem Mater. 2023 Jun 20;35(13):4979–87. doi: 10.1021/acs.chemmater.3c00307 (PMC10339451; doi:10.1021/acs.chemmater.3c00307)
Supplement: Supplementary file 1 — cm3c00307_si_001.pdf [file cm3c00307_si_001.pdf]

Supplementary information: O3 to O1 phase transitions in highly delithiated NMC811 at elevated temperatures

Zachary Ruff[a,b], Chloe S. Coates[a,b], Katharina Märker[a,b], Amoghavarsha Mahadevegowda[b,c], Chao Xu[a,b], Megan Penrod[a,b], Caterina Ducati[b,c], Clare P. Grey[a,b*]

[a]Yusuf Hamied Department of Chemistry, University of Cambridge, Cambridge CB2 1EW, U.K.
[b]The Faraday Institution, Quad One, Harwell Science and Innovation Campus, Didcot OX11 0RA, U.K.
[c]Department of Materials Science and Metallurgy, University of Cambridge, 27 Charles Babbage Road, Cambridge, CB3 0FS, UK

Electrochemical profiles during cell formation and voltage holds

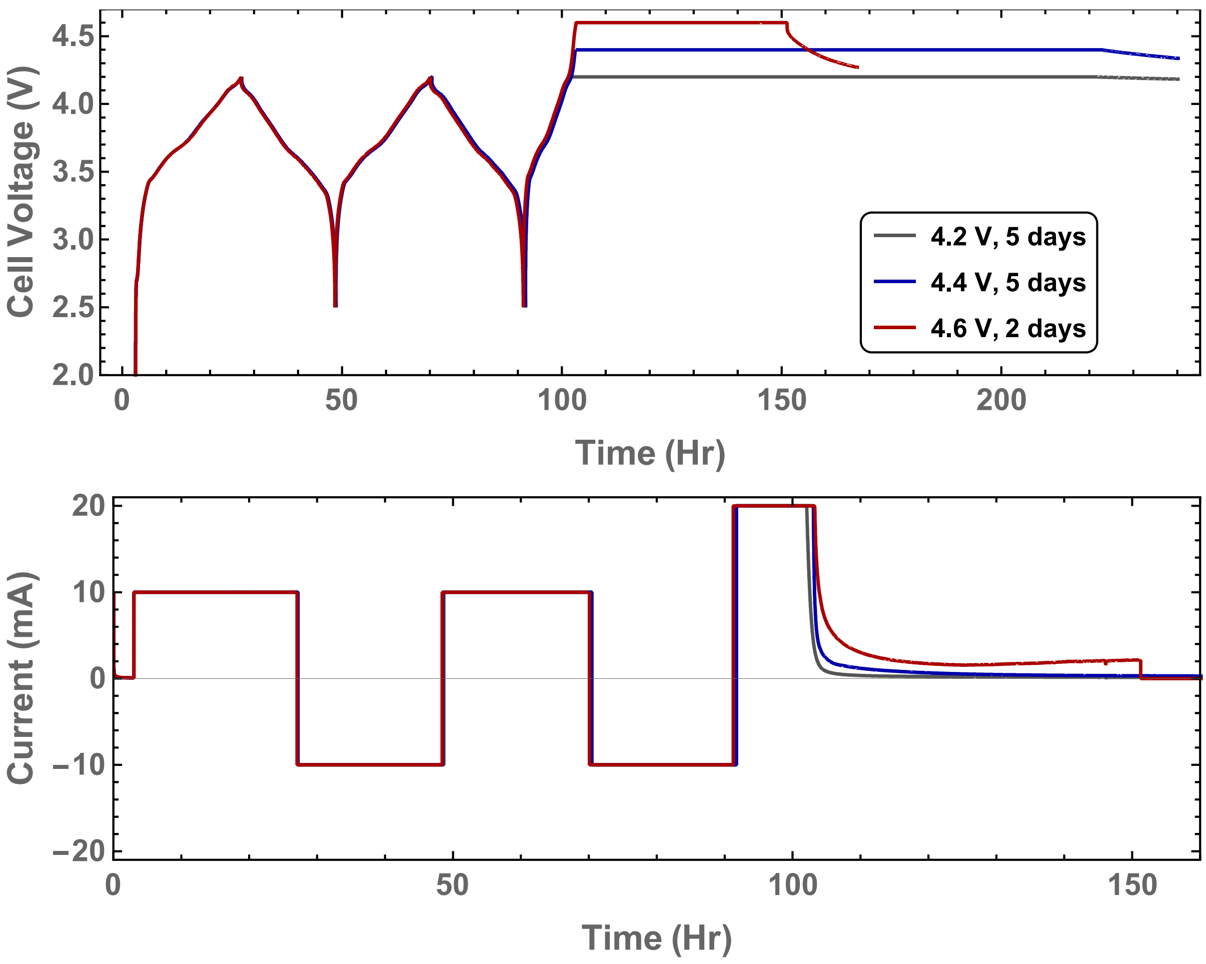

Figure S1. Voltage and current profiles for cells held at 4.2 V for 5 days, 4.4 V for 5 days and 4.6 V for 2 day at 60 °C. The cells first were underwent two formation cycles between 4.2 V and 2.5 V before being held at the upper cut-off voltage.

Table S1. Hold conditions, electrochemical data, ICP measurements and refinement results.

**Hold conditions**			**Electro-chemistry**	**Cathode composition** $Li_x(Ni_{0.8}Mn_{0.1}Co_{0.1})O_2$		**O3 phase**			**O1 phase**			**Refinement FOM****
Voltage (V)	Time (days)	Temp. (ºC)	Total current during hold (mAh/g)	*x*	Adjusted *x* in O3*	Ø (%)	a (Å)	c (Å)	Ø (%)	*a* (Å)	*c* x 3 (Å)	Rwp
Formed, discharged to 2.5 V			-	0.93	-	-	-	-	0	-	-	-
Formed, charged to 4.2 V			-	0.22	-	-	-	-	0	-	-	-
4.2	5	RT	27.5	0.19	-	100	2.817(2)	13.99(2)	0	-	-	4.85889
4.2	5	60	42.7	0.17	-	100	2.839(2)	14.39(2)	0	-	-	6.36743
4.4	5	60	50	0.10	-	100	2.816(2)	13.582(2)	0	-	-	6.34054
4.4	10	60	64.3	0.08	-	100	2.819(0)	13.582(1)	0	-	-	5.06607
4.4	20	60	77.7	0.06	0.07	87	2.817(4)	13.51(1)	13	2.817(0)	13.274(1)	5.64956
4.4	40	60	74.0	0.05	0.07	76	2.817(3)	13.4(4)	24	2.816(0)	13.231(1)	7.00547

**Hold conditions**			**Electro-chemistry**	**Cathode composition** $Li_x(Ni_{0.8}Mn_{0.1}Co_{0.1})O_2$		**O3 phase**			**O1 phase**			**Refinement FOM****
Voltage (V)	Time (days)	Temperature (ºC)	Total current during hold (mAh/g)	*x*	*x* in O3*	Ø (%)	*a* (Å)	c (Å)	Ø (%)	*a* (Å)	*c* x 3 (Å)	Rwp
4.5	5	60	80.8	0.05	0.6	90	2.828(1)	13.630(3)	10	2.822(0)	13.323(3)	4.24208
4.6	2	40	37.8	0.14	0.15	95	2.821(0)	13.499(2)	5	2.817(0)	13.296(3)	5.47861
4.6	0.5	60	44.0	0.09	-	100	2.816(0)	13.710(3)	0	-	-	7.28597
4.6	1	60	61.4	0.06	0.06	94	2.823(0)	13.581(2)	6	2.819(0)	13.308(3)	5.66010
4.6	2	60	103.8	0.06	0.07	93	2.823(0)	13.609(1)	7	2.817(0)	13.299(3)	6.12397
4.6	2	60	108.9	0.08	0.09	90	2.819(0)	13.681(1)	10	2.817(0)	13.303(3)	3.86766
4.6	2.5	60	128.3	0.09	0.10	89	2.817(0)	13.772(4)	11	2.816(0)	13.301(2)	5.32594
4.6 V, 2 days, 60 ºC, discharged			-	0.71	-	100	2.822(4)	13.55(2)	0	-	-	6.44711
4.6 V, 2 days, 60 ºC, 1week at OCV			-	0.21	0.23	100	2.819(1)	14.337(9)	8	2.816(0)	13.364(3)	3.65148
4.7	2	25	46.0	0.03	0.03	95	2.822(0)	13.552(3)	4	2.818(0)	12.933(6)	4.39325
4.7	2	40	64.9	0.03	0.03	100	2.827(0)	13.502(8)	0	2.817(0)	13.302(7)	4.22749

4.8	2	25	74.0	0.05	0.05	95	2.824(0)	13.552(3)	*5*	2.820	13.285	3.85534

*x in O3 phase calculated as described in the experimental section
**Figure of Merit
***2 samples were held at 4.6 V, 60 ºC for 2 day
****Error for lattice parameters is estimated standard deviation of fit

Diffraction patterns graphite anodes

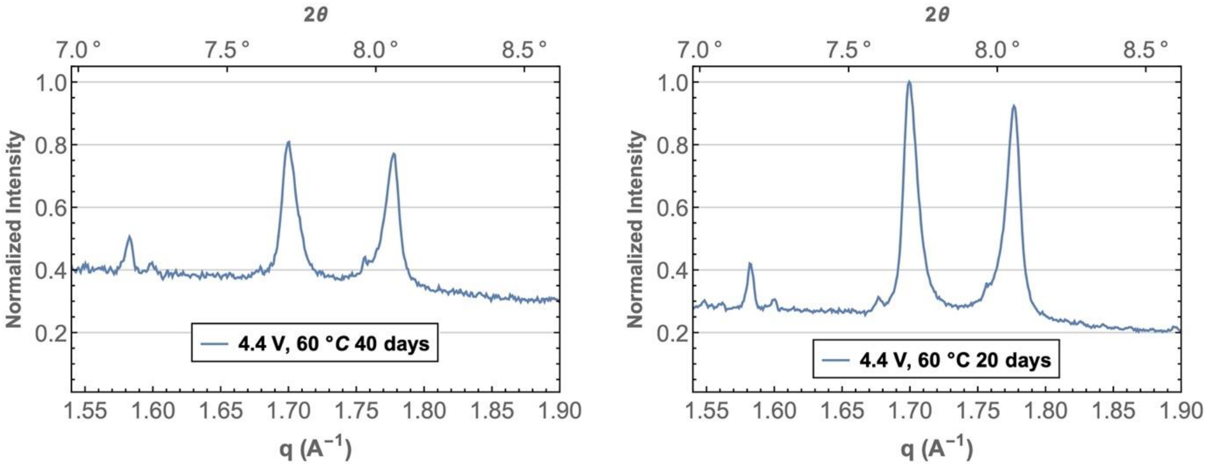

Figure S2. High resolution diffraction patterns (25 keV) from anodes extracted from held cells. The reflection around 1.7 A^{-1} is due to the stage 1 graphite, which has a potential of 70 mV v. Li/Li^{+}.

Diffraction patterns from cells held at 25 °C and 40 °C

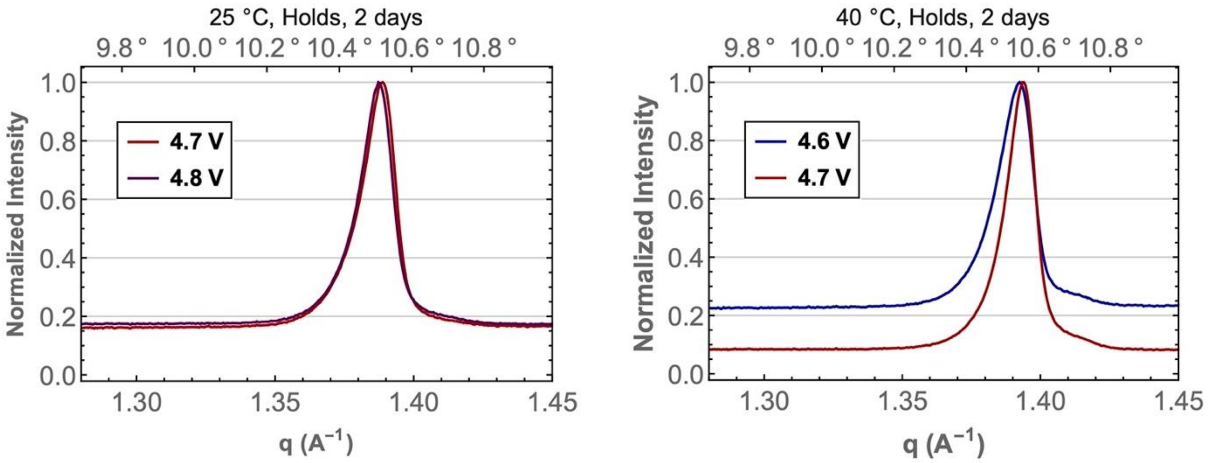

Figure S3. Diffraction patterns from cathodes extracted from cells held at 25 °C (a) and 40 °C (b) for 2 days.

Adjusted lithium concentrations based on no lithium in the O1 phase

$$Li_{x,total} = \emptyset_{O3}\, Li_{x,O3} + \emptyset_{O1}\, Li_{x,O1}$$

$$\text{if } Li_{x,O1} = 0\,, \text{ then } Li_{x,O3} = Li_{x,total}\, \emptyset_{O3}$$

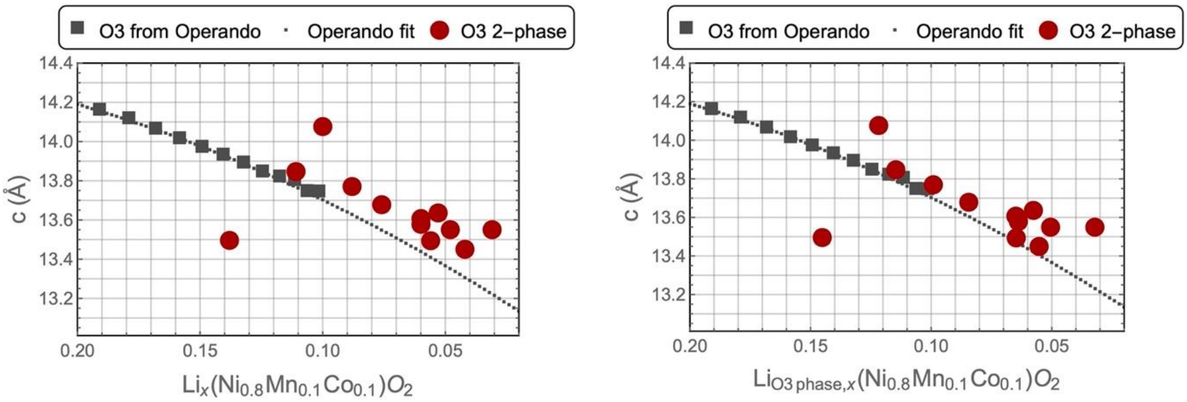

Figure S4. Plot of c-lattice parameter vs. the average lithium concentration in the cathode (left). Plot of c-lattice parameter vs. the adjusted lithium concentration in the cathode assuming that there is no lithium in the O1 phase (right), which is in better agreement with the extrapolated curve from the operando measurements.

Voltage profile of cell held at 4.6 V, 60 °C for 2 days and then left at OCV for 1 week

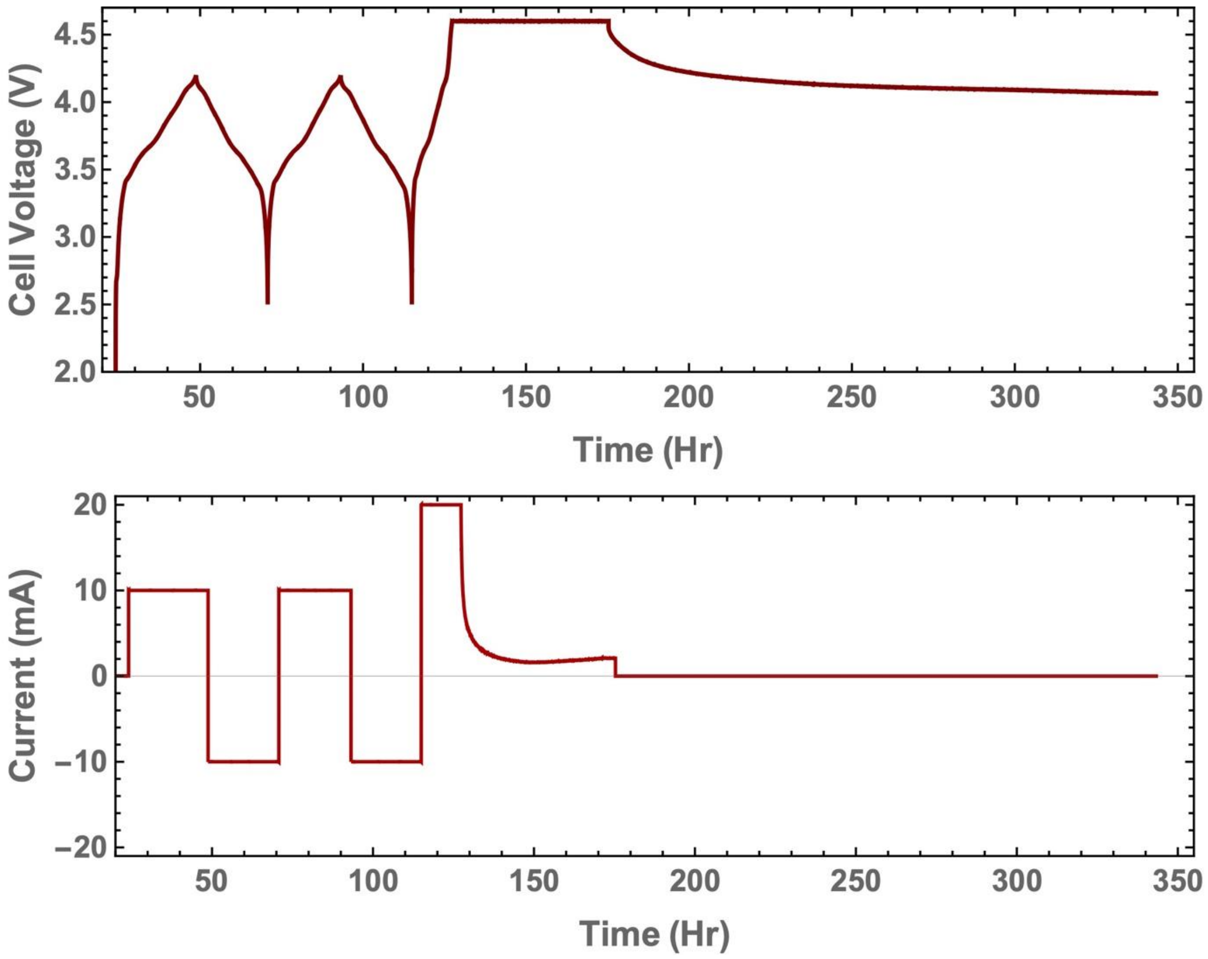

Figure S5. Voltage (top) and current (bottom) profiles from cell that was formed and then held at 4.6 V, 60 °C for 2 days and then left at OCV for 1 week. After 1 week, the cell self-discharge to a 4.06 V before it was disassembled for the diffraction measurement.

Additional solid-state NMR spectra

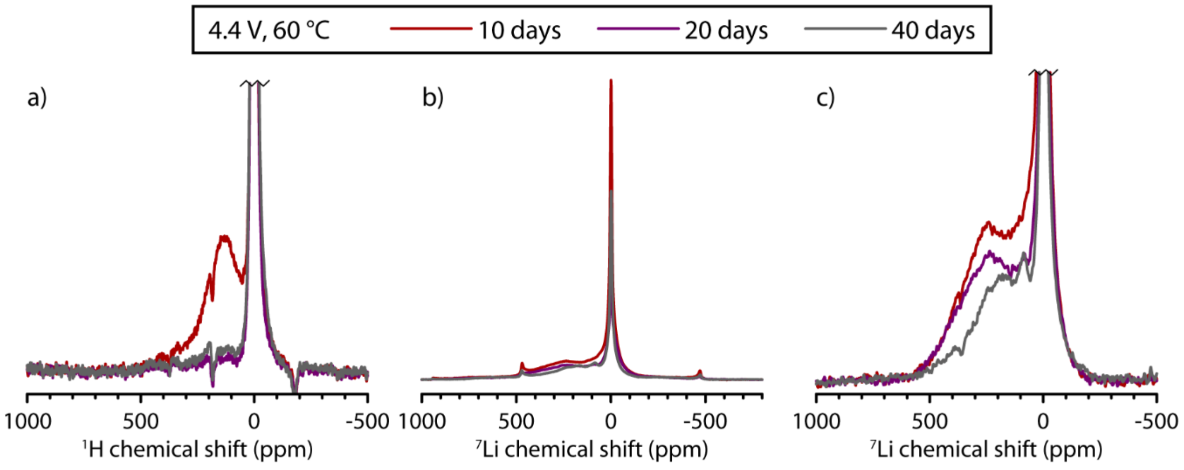

Figure S6. Solid-state NMR spectra of cathodes extracted from cells held at 4.4 V and 60 °C for 10, 20 and 40 days, complementing Figure 5. (a) Central slices of ^{1}H projection-MATPASS spectra.[9] Eight t_1 increments were recorded, with 35840 transients per t_1 increment and a recycle delay of 5 ms. The dips at +378, +185, and -178 ppm are artefacts, presumably resulting from spinning instabilities. (b) Full intensity of the ^{7}Li Hahn echo spectrum shown in Figure 5b. (c) Central slices of ^{7}Li projection-MATPASS spectra. Eight t_1 increments were recorded, with 20480 transients per t_1 increment and a recycle delay of 50 ms. All spectra were recorded at 7.05 T magnetic field strength and 55 kHz magic-angle spinning frequency and are normalized by sample weight and number of scans.

Diffraction pattern from "single-crystal" cathodes

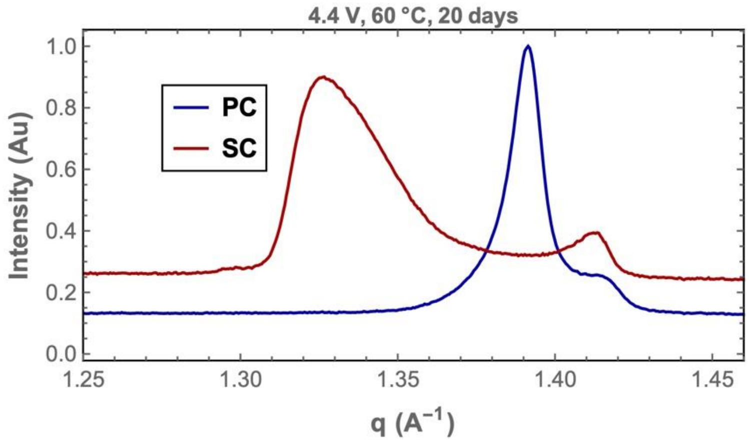

Figure S7. Diffraction patterns from poly-crystal (PC) and "single-crystal" (SC) cathodes held at 4.4 V, 60 °C for 20 days. The composition of the SC cathode is $Li_xNi_{0.83}Mn_{0.1}Co_{0.07}O_2$ rather than $Li_xNi_{0.8}Mn_{0.1}Co_{0.1}O_2$ for the PC cathode.

Indexed simulated diffraction patterns

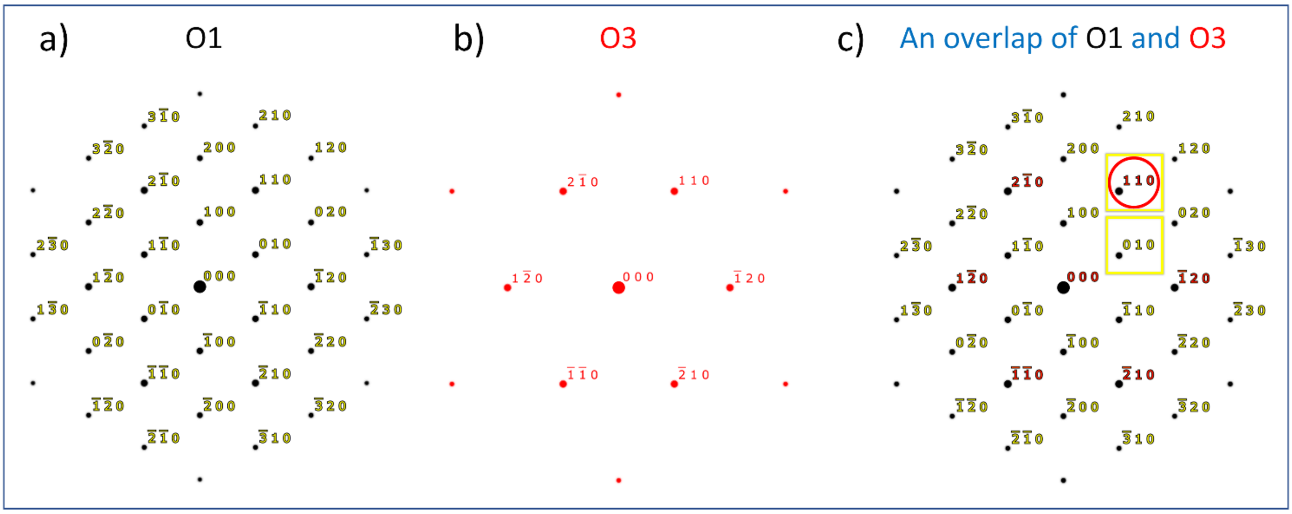

Figure S8. Simulated electron diffraction patterns (with indices) of O1 (a) and O3 (b), and an overlap if these two patterns (c) along the [001] orientation. The simulated patterns (a, b and c) are rotated by 90° counter-clockwise about the out-of plane axis to aid the eye in comparing them with the experimental pattern in Figure 2b..
